# Interactions between the non-seed region of siRNA and RNA-binding RLC/RISC proteins, Ago and TRBP, in mammalian cells

**DOI:** 10.1093/nar/gku153

**Published:** 2014-02-20

**Authors:** Tomoko Takahashi, Shuhei Zenno, Osamu Ishibashi, Toshihiro Takizawa, Kaoru Saigo, Kumiko Ui-Tei

**Affiliations:** ^1^Department of Biophysics and Biochemistry, Graduate School of Science, University of Tokyo, 7-3-1 Hongo, Bunkyo-ku, Tokyo 113-0033, Japan, ^2^Department of Biotechnology, Faculty of Engineering, Maebashi Institute of Technology, 460-1 Kamisadori-cho, Maebashi-shi, Gunma 371-0816, Japan and ^3^Department of Molecular Medicine and Anatomy, Nippon Medical School, 1-1-5 Sendagi, Bunkyo-ku, Tokyo 113-8602, Japan

## Abstract

Small interfering RNA (siRNA)-based RNA interference (RNAi) is widely used for target gene silencing in various organisms. We previously showed that 8-nt-long 5′ proximal nucleotides, which include seed sequence (positions 2–8 from the 5′ end of guide strand), and the complementary sequence of the passenger strand are capable of being simultaneously replaced with cognate deoxyribonucleotides without any substantial loss of gene silencing. In the present study, examination was made of RNA requirements in the non-seed region of siRNA. The non-seed region of siRNA was found to be subdivided into four domains, in which two nucleotide pairs (positions 13 and 14) were replaceable with cognate deoxyribonucleotides without reducing RNAi activity. However, RNA sequences at positions 9-12 and 15-18 were essential for effective gene silencing, and these two double-stranded RNA cores are required for binding of the *trans*-activation response RNA-binding protein (TRBP). The terminal RNA (positions 19–21) provided Argonaute protein binding sites. Argonaute binding was enhanced by the presence of RNAs at positions 15–18. Knockdown experiments showed that, unlike Argonaute and TRBP, Dicer was dispensable for RNAi. Based on these observations, we discuss possible RNA/protein and protein/protein interactions in RNA-induced silencing complex formation.

## INTRODUCTION

RNA interference (RNAi) is a phenomenon whereby double-stranded RNA (dsRNA) leads to posttranscriptional gene silencing through base-pairing interaction (1). RNAi and related RNA silencing mechanisms occur in a variety of evolutionarily diverse organisms (2). In nonmammalian cells, long dsRNAs, which trigger the interferon response in mammalian cells (3,4), are digested by the RNase III endonuclease Dicer (Dcr) to 21–22 nucleotide (nt) small interfering RNA (siRNA) helices with 2-nt 3′ overhangs (5,6). Transfected siRNAs are also capable of triggering RNAi (7). RNAi is directed by the siRNA guide strand acting in concert with several proteins in the RNA-induced silencing complex (RISC) (8). RISC has been purified from fly and human cells mainly using immunoaffinity purification of Argonaute (Ago) protein. As with *Drosophila*, mammalian RISC is formed through dynamic changes in protein–protein and protein–RNA interactions in RISC-loading complexes (RLCs) (9), but the precise mechanism of *in vivo* maturation processes of RISC in mammalian cells remain to be elucidated.

Human RLC may include Ago2, Dcr and dsRNA binding proteins such as *trans*-activation-responsive RNA binding protein (TRBP) as major components (10–12). The RLC for microRNA (miRNA)-based RNAi may include a precursor miRNA (pre-miRNA) hairpin, which is presumed to be processed within the complex during the RISC formation (10). Mammalian RLC activity may be functionally similar to that observed in *Drosophila* embryonic extracts (13).

Dcr was initially determined to catalyze processing of long dsRNA to siRNA units and pre-miRNA to mature miRNA ∼22 bp in length (14–16). However, increasing evidence suggests that Dcr functions not only in the initiation phase (siRNA/miRNA generation), but also in the downstream step in the effector phase, modulating posttranscriptional gene silencing (11,17–19). The coupling of the two phases of RNAi was established by the identification of Dcr-interacting, dsRNA-binding proteins, R2D2 in *Drosophila* (20), Rde-4 in *Caenorhabditis elegans* (21,22), whose human counterparts are thought to be TRBP (11) and a protein activator of PKR (PACT) (23). Dcr alone can process dsRNA and pre-miRNA (11,24–26), but Dcr appears to form a heterodimer with TRBP or PACT (27). TRBP has been reported to significantly enhance Dcr-dependent dsRNA processing (28). Recruitment of siRNAs/miRNAs to Ago may require these Dcr-interacting, dsRNA-binding proteins (11). However, it was revealed recently that Dcr is not always necessary for processing of miRNA and short-hairpin RNAs (shRNAs). Dcr-independent processing of the pre-miRNA-451 (29–31) or shRNA of minimal size (32) has been reported.

Ago2 is the endonuclease of human RISC, and cleaves target mRNA whose sequence is complementary to the guide strand of siRNA. Ago2 may be sufficient for loading and accurate guide strand selection in the absence of Dcr (33–35). In fact, minimal RISC that is capable of cleaving target RNA has been reconstituted using single-stranded siRNA and recombinant Ago2 (36). Furthermore, a recent experiment indicated that the recombinant human Ago2 alone can acquire a guide RNA strand by first associating with siRNA or miRNA duplex, leading to cleavage of target RNA *in vitro* (37), although recombinant Ago2 was initially thought to be unable to bind to siRNA duplex (36,38). Active RISCs are shown to be minimally formed by double-stranded siRNA and Ago *in vitro*, but Dcr and TRBP or PACT may be often considered to be essential for siRNA strand selection (37).

In humans, there are four different Agos (Ago1∼4) highly similar in amino acid sequence to one another (17). Only Ago2 associates with target RNA cleavage activity (39). Unlike *Drosophila* containing two Ago proteins, Ago1 and Ago2, onto which siRNA or miRNA are loaded, mammalian siRNA and miRNA are not differentially sorted by Agos. Ago is composed of four domains: N-terminal (N), Piwi/Argonaute/Zwille (PAZ), Middle (MID), P-element-induced wimpy testis (PIWI) (40). An RNase H-like active site situated in the PIWI domain catalyzes the cleavage of target mRNAs (41–43). N domain is presumed to be involved in RNA unwinding (44). The 5′ end of siRNA is anchored within the binding pocket in the MID domain (45), while the 3′ end is anchored in the PAZ domain (46–48). The current understanding of a mechanistic issue supports a ‘two-state’ model wherein both ends of the guide strand are anchored in the pockets of MID and PAZ domains, respectively, during the nucleation step of target recognition as previously described. However, while its 5′ end is stably anchored in the MID domain, its 3′ end is released from the PAZ pocket owing to topological constraints, following propagation of the duplex toward the 3′ end of the guide strand (8).

TRBP consists of three domains similar in amino acid sequence, two of which (dsRBD1 and dsRBD2) are responsible for RNA binding. The remaining C-terminal domain (dsRBD3) is considered to be in charge of binding to Merlin, Dcr and PACT, but not dsRNA (49). In *Drosophila*, photo-crosslinking studies indicated that the Dcr-2/R2D2 heterodimer determines which siRNA strand associates with Ago and serves as a guide strand (50). Dcr-2 is presumed to bind to the thermodynamically less stable siRNA-end, whereas R2D2 binds to more stable end before loading onto Ago2. In a previous experiment, we suggested that Dcr-free TRBP rather binds to the double-stranded trunk portion of siRNA (51).

In the present study, we systematically introduced 2–3 bp DNA substitutions in the non-seed-duplex region of siRNA with a dsDNA-substituted seed duplex, and identified two subdomains that cannot undergo DNA substitutions without a profound loss of RNAi activity. We found the RNA subdomain capable of enhancing the binding of the Ago-PAZ domain to the terminal non-seed region of siRNA. We also showed that both of these dsRNA cores are essential for effective binding of Dcr-free TRBP to siRNA. RNAi based on siRNA with a dsDNA-substituted seed duplex was found to be completely dispensable for Dcr. These results enable therapeutic approaches for harnessing the untapped potential of RNAi based on RNA–protein interaction.

## MATERIALS AND METHODS

### Cell culture

Chinese hamster ovary CHO-K1 (RIKEN Cell Bank) and human HeLa cells were cultured in Dulbecco’s Modified Eagle’s Medium (DMEM; Gibco BRL) at 37°C. Media for both cell lines were supplemented with 10% heat-inactivated fetal bovine serum (Mitsubishi Kagaku) and antibiotics (10 units/ml penicillin (Meiji) and 50 µg/ml streptomycin (Meiji). Mouse embryonic stem (ES) cells E14TG2a were cultured in DMEM supplemented with 20% heat-inactivated fetal bovine serum (Hyclone), 0.1 mM 2-mercaptoethanol (Wako), 8 µg/ml adenosine, 8.5 µg/ml guanosine, 7.3 µg/ml cytidine, 7.3 µg/ml uridine, 2.4 µg/ml thymidine, 0.1 mM each nonessential amino acid and 1000 units/ml leukemia inhibitor factor (Chemicon International). *Drosophila* S2 cells were cultured in Schneider’s *Drosophila* medium (Gibco BRL) at 25°C.

### Preparation of siRNA and DNA-substituted siRNA

Passenger- and guide-strand RNA or DNA-substituted RNA oligonucleotides were chemically synthesized (Sigma), mixed in a 1:1 fashion in 10 mM NaCl and 20 mM Tris–HCl (pH 7.5), and annealed by incubation at 95°C for 15 min, 37°C for 30 min and 25°C for 30 min. Annealed products were examined using 3% agarose gel electrophoresis in Tris-borate-EDTA buffer, which can separate 21-bp long double-stranded siRNA from 21-nt long single-stranded RNA. Almost all RNA was recovered as dsRNA.

### Gene silencing activity assay for firefly luciferase genes

One milliliter of a CHO-K1 (3 × 10^5^ cells/ml), HeLa (1 × 10^5^ cells/ml) or E14TG2a (2 × 10^5^ cells/ml), S2 (1 × 10^6^ cells/ml) cell suspension was inoculated in a 1.5-cm well 24 h before transfection. Cells were transfected with pGL3-Control DNA (1 or 0.5 µg; Promega) or pGL2-Control DNA (1 µg; Promega) encoding the firefly luciferase (*luc*) gene and pRL-SV40 DNA (0.1 µg; Promega) encoding the *Renilla luc* gene with or without siRNA or DNA-substituted siRNA against the firefly *luc* gene. Knockdown of each protein was performed by transfection of specific siRNA for each protein designed by siDirect2.0 (52–54) 24 h before transfection of the *luc*-encoding vectors. Lipofectamine 2000 reagent (Invitrogen) was used for transfection for CHO-K1, HeLa and E14TG2a cells, and calcium phosphate precipitation method was used for transfection for S2 cells. Cells were harvested 24 h after transfection, and *luc* activity was measured using the dual-luciferase reporter assay system (Promega). In this system, two *luc* (firefly and *Renilla*) genes were expressed simultaneously in cells. The reduction of firefly *luc* activity by the addition of siRNA against the firefly *luc* gene was normalized to the *Renilla luc* activity (internal control).

### Electrophoresis mobility shift analysis

Amplification of the coding sequence of TRBP by polymerase chain reaction (PCR) has been described (51). TRBP protein was produced in *Escherichia coli* BL21 codon plus and Rosetta (DE3) pLysS, as amino-terminal hexa-histidine fusion proteins, and purified with NTA agarose (51). Electrophoresis mobility shift analyses (EMSAs) were performed in binding buffer with 20 mM Tris (pH 7.5), 150 mM NaCl, 2 mM EDTA, 1 mM 2-mercaptoethanol, 1 mM dithiothreitol, 50 ng/ml sonicated salmon sperm DNA and 0.4 U/ml RNasin (Promega). Purified fusion proteins were incubated with siRNA or DNA-substituted siRNA containing ^32^P-labeled guide strand (0.5 nM) for 30 min, and samples were electrophoresed on a 9% polyacrylamide gel and analyzed quantitatively using a FLA-2000 image analyzer (Fujifilm).

For EMSA analysis of the PAZ domain of human AGO, the control pGST-His vector expressing 6xHis and pGST-PAZ-His expressing human Ago1 PAZ domain were constructed. pGST-His was constructed by insertion of annealed oligonucleotides (5′-GGCCGCAGGCAGCAGCCACCACCACCACCACCACTGA-3′ and 5′-GGCCTCAGTGGTGGTGGTGGTGGTGGCTGCTGCCTGC-3′) into the *Not* I site of pGEX-6 P-2 (GE Healthcare). For construction of pGST-PAZ-His, a DNA fragment corresponding to human Ago1 PAZ domain (amino acid residues 222–376) was amplified by PCR with the following primers: 5′-CCGGATCCTTTTATAAGGCACAGCCAGTGATTGAGTTC-3′ and 5′-TCATCAGGGCGGCCGCAGGCAGCAGCCACCACCACCACCACCACTGACTCCTCCTGTCTGTCTGGAGCGGATCTAGC-3′. The amplified fragment was digested with *BamH*I and *Not*I, and inserted into pGST-His. The PAZ domain was expressed in *E. coli* BL21 codon plus (Stratagene) as a glutathione S-transferase (GST) fusion protein, and purified with a glutathione sepharose column (Amersham) according to the manufacturers’ instructions. PAZ protein (1 pmol/µl) was incubated with siRNA or DNA-substituted siRNA containing ^32^P-labeled guide strands (5 fmol/µl each) in 20 mM Tris–HCl (pH 7.5) with 100 mM NaCl, 2 mM EDTA, 1 mM dithiothreitol, 5% glycerol, 50 ng/µl poly(dA-dT) and 2 U/µl RNasein (Promega) for 30 min at room temperature. The mobility shift was analyzed by 5% polyacrylamide gel electrophoresis and quantified using a FLA-2000 image analyzer (Fujifilm).

## RESULTS

### Difference in RNAi properties of an authentic siRNA and dsDNA-substituted siRNA

**Figure 1.**
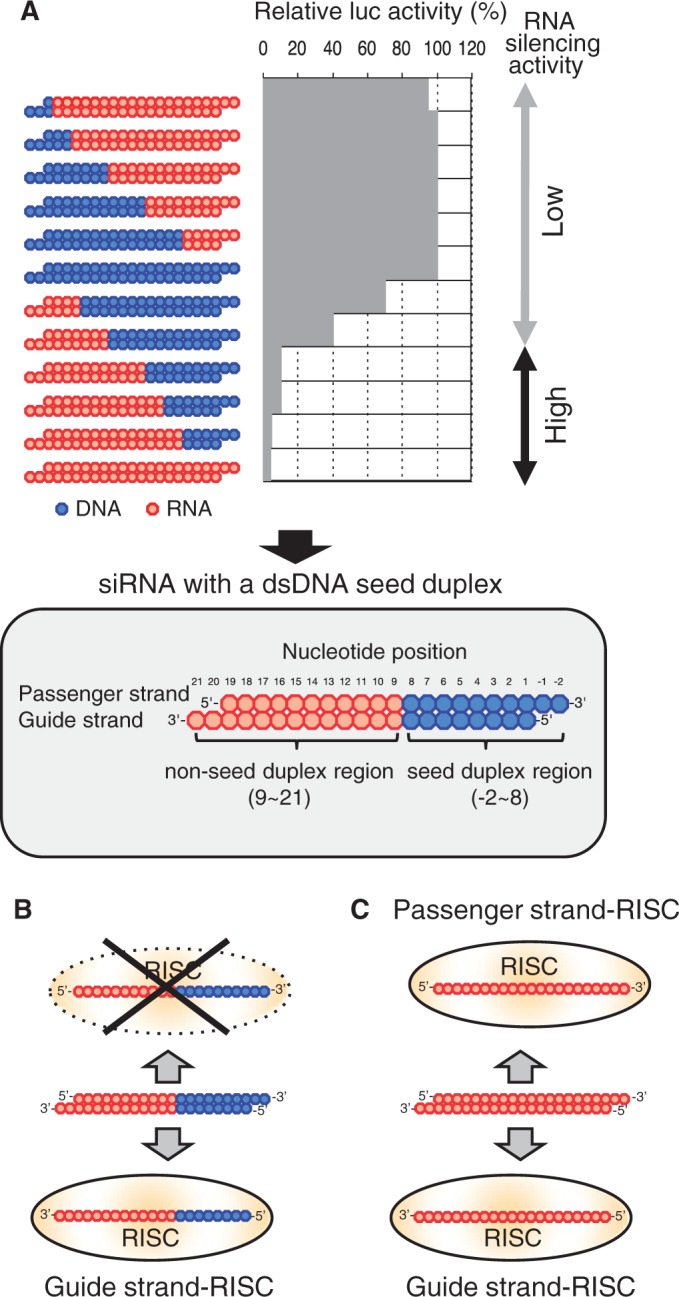
DNA-substitution–dependent changes in target gene silencing due to DNA-modified siRNA (**A**) and asymmetric RISC formation in DNA-modified siRNA-dependent RNAi (**B** and **C**). (A) Effects of dsDNA replacement on RNA silencing activity are shown in the upper panel. The red circle indicates ribonucleotide, whereas blue one indicates deoxyribonucleotide. In each siRNA with or without DNA modification, passenger and guide strands are shown on the top and bottom, respectively. The RNA silencing activity of siRNA with <9 bp DNA substitution from the 5′ end of guide strand is essentially identical to that of nonmodified siRNA (49). Structure of active siRNA with a dsDNA seed duplex, ranging from position 2 to 8, is shown in the lower half. All ribonucleotides in the seed duplex are replaceable with cognate deoxyribonucleotides without any substantial loss of RNAi activity (49). Most, if not all, nucleotides in the remaining (nucleotide position 9–21) should be ribonucleotides, possibly due to binding of RNA-recognition proteins in RLC/RISC. Note that, because of the asymmetry in structure, virtually no off-target effect is associated with siRNA with a dsDNA seed duplex (49). (B) and (C), respectively, show the RISC formation in RNAi due to siRNA with a dsDNA seed duplex and that due to nonmodified siRNA. (B) In the former, the guide strand is recruited to Ago2 to generate a functional RISC but the passenger strand may not be recruited to Ago2 because of asymmetry in structure. (C) In contrast, both guide and passenger strands of the nonmodified siRNA can be recruited to Ago2. Note that the nonmodified siRNA is structural symmetric.

At first, we would like to briefly describe the results of our previous experiment (55), as this study is its natural extension. We constructed siRNAs with various dsDNA substitutions and found that the duplex region ranging from nucleotide position 1 to 8, which includes the guide-strand seed region (nucleotide position 2–8), is replaceable with cognate deoxyribonucleotides without any substantial loss of target gene silencing (Figure 1A) (55). Most part of the remaining non-seed-duplex region could not be replaced with dsDNA (Figure 1A) possibly due to binding of RNA-recognition proteins in the RLC/RISC.

We hereafter referred to the DNA-modified siRNA schematically shown in the lower half of Figure 1A as siRNA with a dsDNA seed duplex and used as a starting material for systematic 2–3 bp dsDNA substitutions carried out in the present experiment.

siRNA with a dsDNA seed duplex was found to be associated with little passenger-strand–dependent gene-silencing activity or seed-dependent off-target effects (55). Thus, in RNAi triggered by siRNA with a dsDNA seed duplex, the guide strand appears to be properly recruited to Agos to generate functional RISCs, while the passenger strand cannot (Figure 1B).

In contrast to siRNA with a dsDNA seed duplex, the authentic siRNA is rotationally symmetric with respect to RNA structure. Because of this, Ago binding may occur in two orientations, resulting in two forms of functional RISCs, one including the guide strand and the other the passenger strand (Figure1C). Balance in formation between these two RISCs is presumed to be determined based on difference in thermodynamic stability of two siRNA ends (43,56,57). Thus, siRNA with a dsDNA seed duplex may provide a much simpler system for biochemical substructure analysis of the non-seed region than the authentic nonmodified siRNA.

### RNA requirements in the non-seed region of siRNA

**Figure 2.**
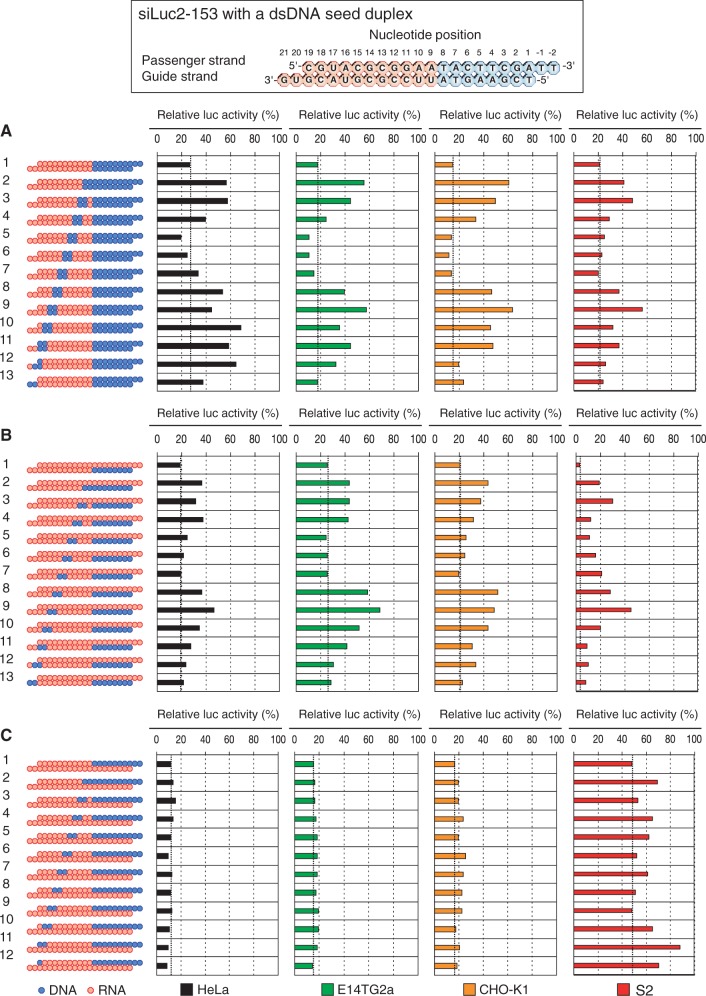
Effects of 2-bp-long DNA substitutions in the non-seed region of siRNA (siLuc2-153) with a dsDNA seed duplex on RNAi activity as determined by *luc* reporter assays. Except for DNA/RNA difference, the nucleotide sequences of DNA-modified siRNAs used here were identical to that of siLuc2-153. Red circle, ribonucleotide; blue circle, deoxyribonucleotide. (**A**) Both guide and passenger strands were simultaneously replaced with DNA. (**B**) DNA replacement was carried out only in the guide strand. (**C**) DNA replacement was carried out only in the passenger strand. RNAi was assayed using human HeLa, mouse E14TG2a, Chinese hamster CHO-K1 and *Drosophila* S2 cells using a dual-luciferase reporter assay system with nonmodified or DNA-substituted siRNAs at 50 nM. However, in S2 RNAi (B), the siRNA concentration used was 5 nM. The dotted line in each graph indicates the level of RNAi due to the parental modified siRNA. Lane 1, siRNA with a dsDNA seed duplex. Lane 2–lane 13, siRNA with a dsDNA-modified seed duplex and two additionally DNA-substituted base pairs in the non-seed-duplex region.

To clarify RNA requirements in the siRNA non-seed region, a 2–3 bp DNA substitution was additionally introduced into siRNA with a dsDNA seed duplex and subjected to RNAi assay using cultured cells. Structures of each siRNA derivative were examined, and changes in RNAi activity observed are shown in Figures 2A and Supplementary Figure S1A. One hundred percent in relative *luc* activity in Figure 2 and Supplementary Figure S1 corresponds to the absence of RNAi activity. We used three mammalian cell lines (human cervical cancer-derived HeLa, mouse ES E14TG2a and Chinese hamster ovary-derived CHO-K1 cells) and *Drosophila* S2 cells for transfection. siRNA sequences used were of highly functional ones against *luc* (52), siLuc2-153 (Figure 2) and siLuc-36 (Supplementary Figure S1). These siRNAs were same siRNAs used in our previous study (55). siRNA concentrations used were 50 or 5 nM. In all cases, position-dependent changes in RNAi activity were similar, if not identical to each other.

**Figure 3.**
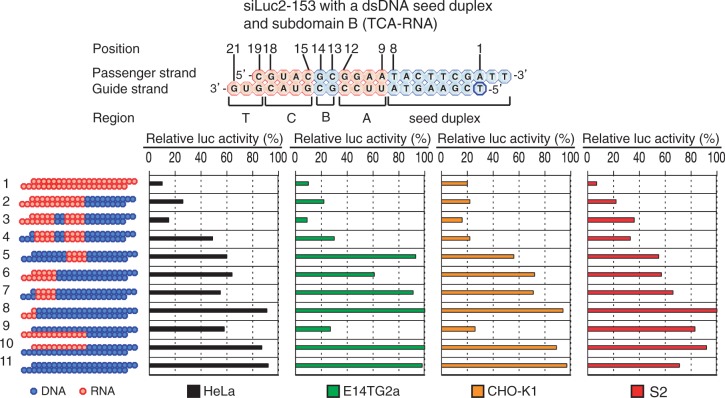
Effects of DNA substitutions in the non-seed-duplex subdomains A, B, C and T on RNAi activity. Except for DNA/RNA difference, the nucleotide sequences of DNA-modified siRNAs were identical to that of siLuc2-153. Red circle, ribonucleotide; blue circle, deoxyribonucleotide. Seed duplex, nucleotide position 2–8; subdomain A, position 9–12; subdomain B, position 13–14; subdomain C, position 15–18; subdomain T, position 19–21. RNAi activity was assayed using HeLa, E14TG2a, CHO-K1 and S2 cells, using a dual-luciferase reporter assay system. The concentration of siRNA was 50 nM. Lane 1, nonmodified siRNA; lane 2, TCBA-RNA (the entire non-seed duplex is RNA); lane 3, TCA-RNA; lane 4, CA-RNA; lane 5, A-RNA; lane 6, TC-RNA; lane 7, C-RNA; lane 8, T-RNA; lane 9, RNA in the guide strand non-seed region; lane 10, RNA in the passenger non-seed region; lane 11, siDNA.

DNA substitutions at positions 13 and 14 and of the 3′ overhang of the guide strand changed little, if any, RNAi activity (lanes 6, 12, 13). DNA replacement at nucleotide position 19 gave some RNAi activity reduction in a cell-type–dependent manner (lanes 13): gene silencing activity was considerably reduced in HeLa and E14TG2a cells but that in CHO-K1 and S2 cells was not significant. If not otherwise mentioned, the 3′ overhang of the guide strand and the nucleotide pair at position 19 are collectively called the subdomain T. Based on these observations, we considered that the non-seed region of siRNA are divided into four subdomains and that two of them, A (from nucleotide position 9 to 12) and C (from position 15 to18) subdomains, should be RNA for active RNAi (see the upper margin of Figure 3 and Supplementary Figure S2). Subdomain B corresponds to two nucleotide pairs at position 13 and position 14. Subdomain B is replaceable with DNA.

Figure 2B and Supplementary Figure S1B show that *luc* gene silencing due to a 2–3 bp dsDNA substitution is strongly correlated with those induced by a similar DNA substitution only in the guide strand. In contrast, transfection with siRNAs with DNA substitution only in the passenger strand gave a small, if any, effect on *luc* target gene silencing in mammalian cell (Figure 2C and Supplementary Figure S1C). Note that all nucleotides of the passenger and guide strands, respectively, in Figure 2B and C (or Supplementary Figure 1B and C) are ribonucleotides. Although these results may indicate that RNA requirement in the non-seed region is due primarily to interactions with RLC/RISC protein moieties, it should be noted that 2–3-bp DNA substitutions have never completely abolished relative *luc* activity (Figure 2 and Supplementary Figure S1), possibly suggesting that either 2–3-bp-long DNA segments are too short to completely suppress RNAi or there is some ambiguity in positioning of putative RNA-binding proteins in RLC/RISC.

The contribution of the passenger strand to gene silencing is much greater in *Drosophila* than mammalian cells (Figure 2C and Supplementary Figure S1C). However, this is not due to the effect of introduction of an additional 2–3-bp DNA substitution in the non-seed region, as virtually no difference in gene silencing activity between parental and additionally DNA-modified siRNAs (compare lane 1 and other lanes in Figure 2C or lane 1 and other lanes in Supplementary Figure S1C).

### DNA-substitution–induced inactivation of three RNA-requiring subdomains found in the non-seed-duplex region of siRNA

To make certain that the siRNA non-seed-duplex region consists of four subdomains (see the upper margins of Figure 3 and Supplementary Figure S2), various DNA substitution variants were constructed and their RNAi-inducing activity was examined (Figure 3 and Supplementary Figure S2). Lane 1 and lane 2 are positive controls, indicating RNA in the entire non-seed-duplex region to give a highly active RNAi activity. Lane 11 is a negative control and the relative *luc* activity induced was virtually 100%, indicating no or little functional RNAi to be induced by siRNA in which all ribonucleotides are replaced with cognate deoxyribonucleotides (siDNA). If necessary, DNA substitution variants are hereafter simply referred to as ‘an RNA domain combination’. Thus, for example, TC-RNA means a DNA-substitution variant of siRNA in which only subdomains T and C are RNA.

As expected, the introduction of DNA into subdomain B (TCA-RNA) resulted in only a slight, if any, reduction of RNAi activity (lane 3), indicating that RNA in subdomain B is not essential for functional RNAi in all cell types examined. DNA substitutions in both subdomains B and T (CA-RNA) (lane 4) induced a considerable reduction of RNAi in human and *Drosophila* cells but not in mouse and Chinese hamster cells, suggesting that RNAi activity due to DNA substitution in subdomain T to occur in a cell-type–dependent manner. We interpret these findings as suggesting the presence of some difference in siRNA binding mode among three mammalian and *Drosophil*a cells during RLC/RISC development.

RNA only in subdomain A (A-RNA; lane 5) or only in subdomain C (C-RNA; lane 7) could not trigger effective RNAi, whereas the introduction of RNA into subdomain T to the C-RNA derivative slightly improve RNAi activity in particular in the cases of siLuc-36 derivatives (lane 6 in Supplementary Figure S2). But RNA in subdomain T could not solely recover the abolished RNAi activity at all (compare lane 8 and lane 11). Thus, we consider that synergistic interactions among three RNA subdomains A, C and T may be important for maintaining siRNA-based RNAi activity.

In the above section, we showed that the guide strand is much more important to the DNA-dependent suppression of RNAi activity than the passenger strand. To further extend this notion, two types of DNA-modified siRNA containing a dsDNA seed duplex were constructed. In one of them, DNA substitution was introduced only in the four subdomains of the passenger strand (lane 9), while in the other, the guide strand domains (lane10). Again, we noted the guide strand preference. In fact, a partial and cell-type–dependent RNAi activity was found to be associated with the presence of RNA in the non-seed-duplex guide strand but not the non-seed-duplex passenger strand.

### Involvement of Ago and TRBP but not Dcr in human RNAi due to siRNA with a dsDNA seed duplex

**Figure 4.**
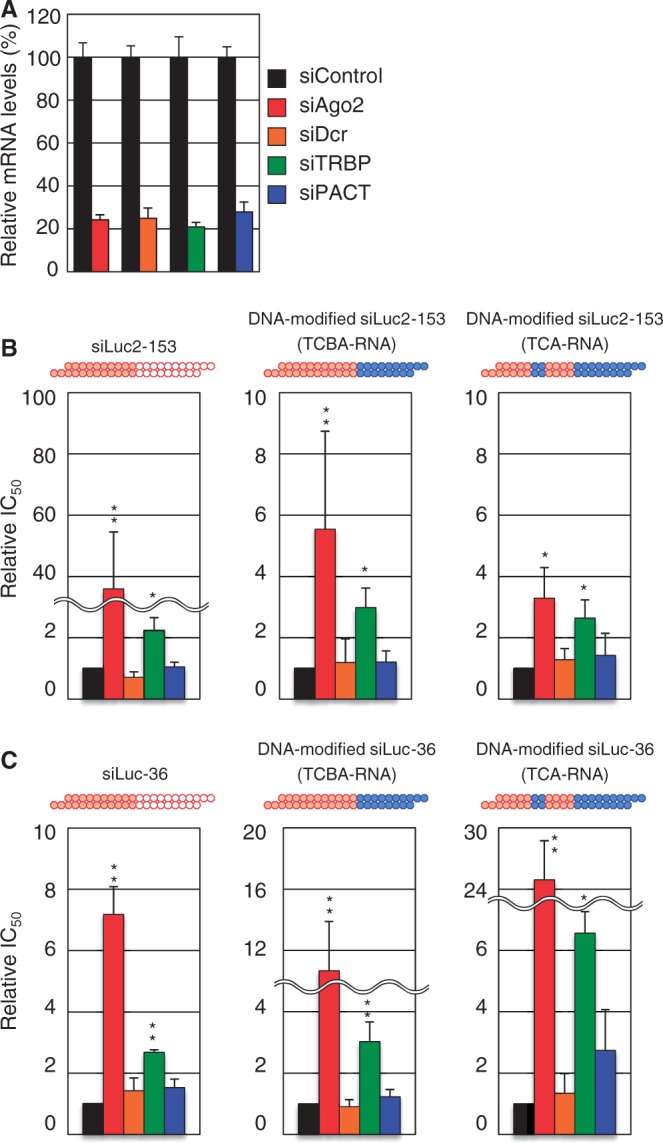
Effects of Ago2, Dcr, TRBP and PACT knockdown on *luc* RNAi due to DNA-modified or nonmodified siRNA. Except for DNA/RNA difference, the nucleotide sequences of DNA-modified siRNAs were identical to that of siLuc2-153 or siLuc-36 as indicated. Red circle, ribonucleotide; blue circle, deoxyribonucleotide. (**A**) Knockdown efficiency of siAgo2, siDcr, siTRBP or siPACT was examined by real-time reverse transcriptase-PCR in HeLa cells. Changes in expression level of each mRNA normalized with that in the cells treated with siControl were measured following the addition of 25 nM siAgo2, siDcr, siTRBP or siPACT. (**B** and **C**) Relative change in IC_50_s of Ago2, Dcr, TRBP and PACT knockdown of siLuc2-153 (B) and siLuc-36 (C). Relative IC_50_ was normalized using that for RNAi due to control siRNA, siGY441. IC_50_ values were calculated using the dual-luciferase reporter assay data in Supplementary Figures S3 and S4. *P*-values were determined by Student’s *t*-test (**P < 0.01, *P < 0.05).

Human RLC/RISC may include Ago, TRBP, PACT and Dcr as major components (10–12). Thus, examination was made of whether these gene products are functionally required for RNAi due to three different types of anti-*luc* siRNA, which consist of authentic siRNA, siRNA with a dsDNA seed duplex (TCBA-RNA) and siRNA with simultaneously dsDNA-replaced seed-duplex and subdomain B (TCA-RNA) (see the upper margin of Figure 4B and C). siGY441, an siRNA specific to GFP, was used as an siRNA control. As shown in Figure 4A, ∼80% of the expression of any one of RLC/RISC genes examined was found to be abolished on transfection of corresponding siRNA, indicating a successful inactivation of relevant genes to occur.

RNAi activity against a background of RLC/RISC-related gene knockdown was quantitatively analyzed (Supplementary Figures S3 and S4). In each case, the concentration of 50% inhibition (IC_50_) was normalized by that for siGY441 treatment and the resultant relative IC_50_ s were shown for comparison. Although relative IC_50_ values considerably varied depending on target *luc* sequences, it is virtually evident that Ago2 and TRBP are profoundly involved in RNAi due to all three types of siRNAs used and that Ago2 knockdown results in changes much larger than TRBP knockdown (Figure 4B and C). In contrast, siDcr transfection exhibited no or little effect on RNAi activity not only due to DNA-modified siRNA but due to authentic siRNA as well. This finding was further confirmed by transfection of mouse mutant cells lacking Dcr activity (T.T., unpublished observation). Thus, Dcr may not be involved in mammalian RNAi based on 21-bp-long authentic siRNA or those due to DNA-modified siRNAs exhibited here (TCBA-RNA and TCA-RNA). Alternatively, Dcr is involved in mammalian RNAi but totally redundant in function. In fact, there is a body of literature on Dcr-independent processing of miRNAs or shRNAs (29–32). In this connection, it may be noteworthy to point out that Dcr has been shown to be necessary for shRNA-based RNAi, which includes maturation processes of a small RNA component (33).

The PACT-containing complex is shown to enhance the strand selection for some miRNAs and inhibits the processing of pre-siRNA substrates (37). At present we are not yet sure about whether PACT is involved in RNAi due to siRNA with or without a dsDNA seed-duplex substitution, as relative IC_50_ values except for one case (Figure 4C, TCA-RNA) appeared small, if any (Figure 4B and C).

### Ago protein binding to the non-seed duplex is enhanced by subdomain C, one of the central dsRNA subdomains

X-ray crystallographic analysis indicated the PAZ domain of Ago protein to contain a pocket capable of binding to the 3′ end of single-stranded RNA (46–48). However, we are not yet sure about how Ago-PAZ protein domain can recognize all or part of four subdomains in the non-seed duplex of siRNA for binding. In human, there are four Ago paralogs (Ago1∼4) whose PAZ domains are similar in amino acid sequence to one another [(17); Supplementary Figure S5]. Thus, to clarify possible interactions between non-seed-duplex subdomains and the PAZ domain of Ago protein, examination was made of interactions between the PAZ domain of Ago1, tagged with GST, and subdomains of the siRNA non-seed duplex. RNA–protein interaction was examined using EMSA assay.

**Figure 5.**
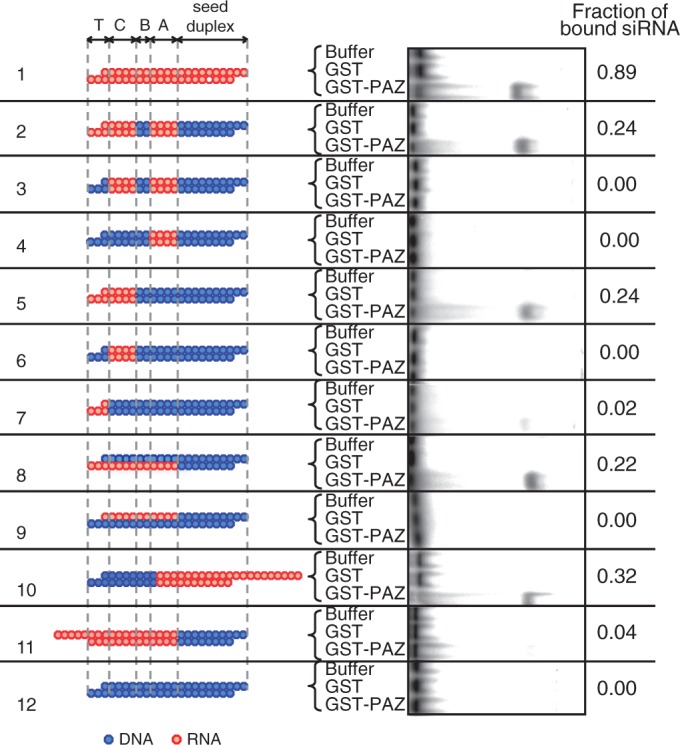
EMSA profiles of DNA-substituted siRNAs associated with recombinant PAZ domain of human AGO1. EMSA experiments were carried out using recombinant human AGO1 PAZ domain with ^32^P-labeled authentic siLuc-36 or its DNA-substituted derivatives. Relative fractions of bound siRNA are shown by numerals on the left. Lane 1, nonmodified siRNA; lane 2, TCA-RNA; lane 3, CA-RNA; lane 4, A-RNA; lane 5, TC-RNA; lane 6, C-RNA; lane 7, T-RNA; lane 8, RNA in the guide strand non-seed region; lane 9, RNA in the passenger non-seed region; lane 10, 10-nt 3′ protrusion of passenger strand; lane 11, 5-nt 5′ protrusion of passenger strand; lane 12, siDNA.

As shown in lane 1 and lane 12 in Figure 5, GST-PAZ was found to be capable of binding to siRNA but not siDNA, suggesting that PAZ can interact with double-stranded siRNA but not siDNA. Subdomain B is an siRNA domain replaceable with DNA without any substantial loss of RNAi activity and hence, is presumed to be unrelated to PAZ binding. In contrast, the three remaining subdomains, A, C and T should be RNA for effective RNAi. In fact, a strong interaction was detected between GST-PAZ and DNA-modified siRNA with RNA in all three subdomains (TCA-RNA; lane 2). We introduced an additional DNA replacement in subdomain T (lane 3) and found that resultant DNA-modified siRNA with RNA in subdomains A, C (CA-RNA) was found to be incapable of binding to GST-PAZ at all. Similarly, all other siRNAs with DNA in subdomain T failed to show any GST-PAZ binding (lane 4, lane 6). Contrary to these, DNA-modified siRNAs with RNA in subdomain T were associated with GST-PAZ binding (lane 2, lane 5, lane7). Thus, we conclude that it is the terminal subdomain T that provides PAZ binding sites.

Lane 5 and lane 7 in Figure 5 showed that GST-PAZ binding activity to increase >10 times when DNA-modified siRNA possesses RNA sequences not only in subdomain T but also in subdomain C (TC-RNA), indicating that subdomain C serves as an enhancer for GST-PAZ binding to subdomain T. As no improvement in GST-PAZ binding activity was detected when subdomain A was of RNA (lane 2), suggesting that, unlike subdomain C, subdomain A possesses little enhancer activity, if any. In a previous section, we showed that, in RNAi due to siRNA with a dsRNA seed duplex, there is a strong preference for the guide strand. Thus, strand preference in interactions between the non-seed region of siRNA and PAZ domain of Ago protein was examined (lane 8 and lane 9). Again, we found that RNA in the guide strand is much more preferable for Ago-siRNA binding than that in the passenger strand. As described above, DNA substitutions in 2-nt 3′ overhang of the guide strand and subdomain A did not significantly change PAZ-siRNA binding. Thus, we conclude that the position-19 nucleotide of the guide strand and RNA sequence in subdomain C of the guide strand, respectively, are primarily important for PAZ binding and enhancer function for PAZ binding to siRNA in human HeLa cells.

Finally, we examined some structural features of siRNA ends for PAZ binding. Lane 10 and lane 11, respectively, showed the results of PAZ binding when 3′ or 5′ protruding RNA were used as the substrates for binding. We found that the end with 5′ protrusion is much less efficient in PAZ protein binding than the end with 3′ protrusion, suggesting that PAZ prefers to a 3′ protruding siRNA end for binding and that 5′ protrusion in the siRNA end may prevent PAZ from interacting with siRNA. Note that DNA substitution in the lane 10 siRNA is opposite in orientation to all other siRNAs shown here.

### Requirements of two central, non-seed-duplex siRNA subdomains, A and C, for effective TRBP binding

**Figure 6.**
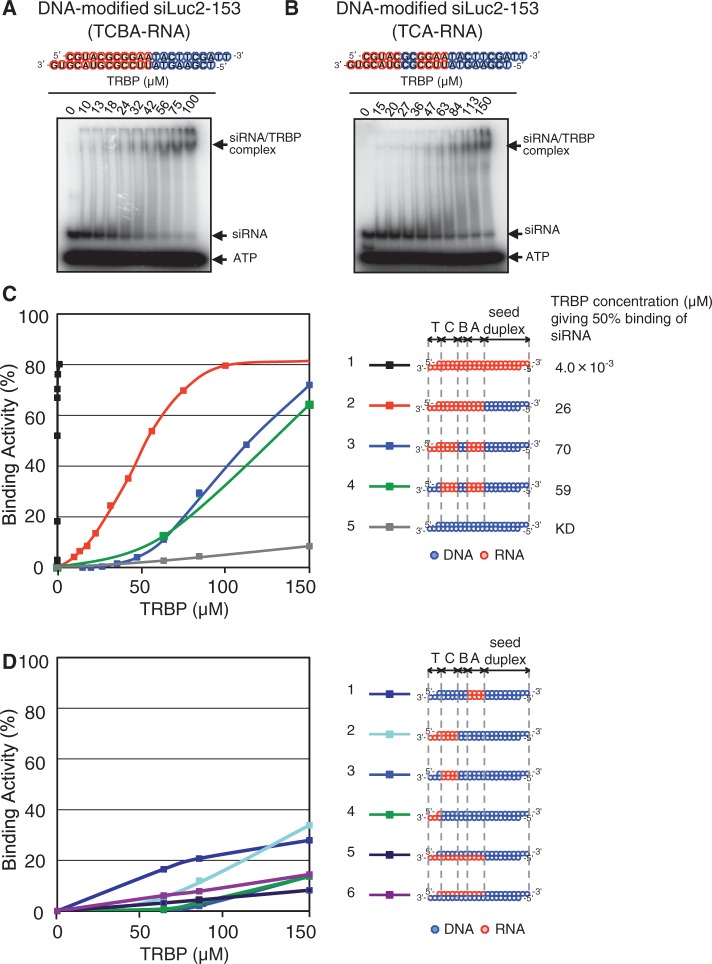
EMSA of DNA-substituted siRNAs associated with recombinant human TRBP protein. Except for DNA/RNA difference, the nucleotide sequence of DNA-modified siRNA was identical to that of siLuc2-153. Red circle, ribonucleotide; blue circle, deoxyribonucleotide. (**A** and **B**) EMSA experiments. Purified recombinant TRBP and ^32^P-labeled siRNA with DNA substitutions (0.5 nM) were incubated with increasing amounts of wild-type TRBP protein, as indicated. In both (A) and (B), seed duplex region was replaced with dsDNA. In (B), dsDNA substitution was also carried out in subdomain B. (**C** and **D**) A bound fraction (%) of siRNA was plotted against the input concentration of TRBP. Note that the binding activities of DNA-modified siRNAs were significantly lower than that of the nonmodified siRNA. Structures of DNA modifications are shown in the right margin (C and D). In (C), observed TRBP concentrations giving 50% binding of nonmodified siRNA and siRNA with a dsDNA seed duplex are also shown in the right margin.

TRBP knockdown experiments may suggest that TRBP contribution to RNAi due to siRNA with a dsDNA seed duplex is similar, if not identical, to that due to nonmodified siRNA (see Figure 4B and C). Our previous experiments showed interactions between recombinant TRBP and authentic siRNA to be carried out in a two-step fashion (51). The first-step complex consists of TRBP monomer and one molecule of authentic siRNA, whereas the second-step complex, TRBP homodimer and siRNA monomer. Here we analyzed putative complex formation between TRBP protein and siRNA with a dsDNA seed duplex using a similar strategy. In both cases, complex formation was analyzed by EMSA assays (Figure 6A and B and Supplementary Figures S6A and B and S7).

At a first glance, TRBP binding to siRNA with a dsDNA seed duplex strikingly differed from that to the authentic siRNA (Figure 6C and Supplementary Figure S6C). As shown previously (51), in the case of nonmodified siRNA binding, the TRBP concentration giving 50% binding of siRNA was estimated at 4.0∼5.3 × 10^−^^3 ^μM, whereas that for siRNA with a dsDNA seed duplex (TCBA-RNA), 15∼26 μM (Figure 6C and Supplementary Figure S6C). Note that the latter is ∼500 times as high as the former concentration. As described in ‘Discussion’ section, we interpret these results as suggesting that TRBP binding to siRNA with a dsDNA seed duplex within cells is not weak, and that only TRBP associated with an unidentified RNA-binding protein ‘X’, which is capable of strong binding to siRNA with a dsDNA seed duplex, can effectively bind to siRNA with a DNA-modified guide sequence.

Next, we examined TRBP capability for binding to four subdomains consisting of the non-seed region of siRNA. As shown in Figure 6C and Supplementary Figure S6C, introduction of DNA substitutions into subdomain B gave somewhat increased TRBP concentration giving 50% binding of siRNA or exhibited a slightly lower affinity to siRNA (compare lane 2 and lane 3), and it appeared slightly increased by an additional DNA replacement in subdomain T (lane 4), suggesting low levels of contribution of RNA in subdomains B and T for TRBP binding. In contrast, as expected, siDNA exhibited virtually no siRNA binding activity (lane 5).

DNA-modified siRNA containing RNA only in one of the non-seed-duplex subdomains other than subdomain B gave much reduced values of the TRBP concentration giving 50% binding of siRNA (lane 1, lane 3 and lane 4 in Figure 6D and Supplementary Figure S6D). siRNAs with RNA in (T+C) subdomains (TC-RNA) also gave low values (lane 2 in Figure 6D and Supplementary Figure S6D). Taken together, these results may indicate that the physical association between TRBP protein and the non-seed duplex of siRNA requires a simultaneous presence of nonterminal subdomains, A and C.

Finally, we examined the effect of guide- and passenger-strand-specific DNA-replacement in the non-seed region on the activity of TRBP binding to siRNA. Consistent with a previous experiment (51), we could find no appreciable difference between them, although TRBP binding was low (lane 5 and lane 6 in Figure 6D and Supplementary Figure S6D).

## DISCUSSION

A highly functional siRNA may consist of two regions, the seed duplex region, which is capable of being totally replaced with DNA without substantial loss of gene silencing activity, and the non-seed-duplex region, most of which should be RNA and possibly provide binding sites for RNA-binding RLC/RISC proteins (55). Gene silencing due to siRNA with a dsDNA seed duplex has been shown to be similar, if not identical, to RNAi due to authentic or nonmodified siRNA (55). Taking advantage of these findings, the present study was carried out and demonstrated the non-seed region of siRNA to consist of four subdomains with respect to RNA requirements not only in mammalian cells such as human HeLa, mouse E14TG2a and Chinese hamster CHO-K1 cells but in *Drosophila* S2 cells as well (see the upper margin of Figure 3). These findings may indicate that subdomain structure found in siRNA is one of the skeletal mechanisms of RNAi.

**Figure 7.**
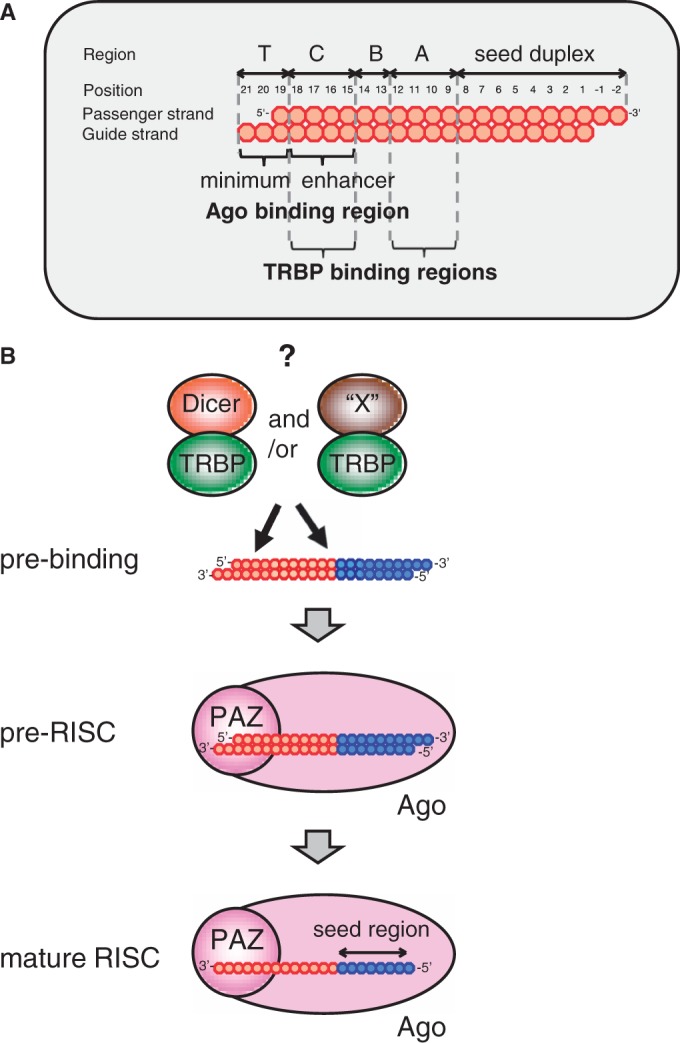
Presumed subdomain structure of siRNA (**A**) and possible models for functional RISC formation (**B**) A deduced subdomain structure of siRNA is shown in (A). Subdomain A is required for TRBP binding. Subdomain B is a region replaceable with DNA without a substantial loss of RNAi activity. Subdomain C is not only required for TRBP binding but also serves as an enhancer for Ago2/siRNA binding. Subdomain T provides Ago2 binding. (B) Possible case of RISC formation is depicted. A model consists of three steps. Pre-binding step includes an unidentified factor, ‘X’, which is presumed to be capable of binding to TRBP and recruiting it to siRNA. Note that one of the possible candidates of ‘X’ might be Ago. Dcr is totally dispensable or redundant in function to ‘X’. Although in some cases, RNAi due to siRNA with a dsDNA seed duplex may include neither ‘X’ nor Dcr. In pre-RISC, DNA-modified siRNA duplex is recognized with the PAZ domain of Ago and, in the mature RISC, consisting of Ago2 and the guide strand, is formed.

Our results (see Figure 5) showed that the terminal subdomain (subdomain T) and its neighbor (subdomain C), respectively, provide a human Ago (PAZ domain) binding site and human Ago binding enhancer function and that RNA in two of the three central subdomains is essential for human TRBP binding (Figure 7A). In contrast to Ago and TRBP, Dcr activity was not required for any step of RNAi, which is triggered by 21-bp-long, highly functional, nonmodified siRNA or siRNA with a dsDNA seed duplex within human cells, indicating that Dcr is redundant in function or is uninvolved in human RNAi examined in the present study. We used two nonoverlapping target sequences of *luc* (targets for siLuc2-153 and siLuc-36) for general value and obtained an identical conclusion.

It is known that the PAZ domain specifically recognizes an RNA with a 2-nt 3′ overhang. Then, if the subdomain T is RNA, this substrate would not be expected to perform a binding defect with Ago-PAZ protein domain. Thus, the mechanistic implications of a weak Ago binding to the DNA-substituted siRNA variant with RNA only in subdomain T (T-RNA) (Figure 5, lane 7) and the enhancer function of subdomain C (Figure 5, lane5) remain unclear. To our knowledge, we are not sure whether PAZ domain is sensitive to differing geometries, one of the possible explanations may be that the regional geometry of subdomain T is perturbed away from a typical A-form RNA duplex toward B-form by the DNA base pairs in the neighboring subdomain C. In the ‘two-state’ model, the MID/PIWI domains have important function in substrate recognition, while the 3′ end is released from PAZ pocket during nucleation steps. Thus, although the PAZ domain is less important to canonical RNAi function, it does not rule out possibility that the PAZ domain playing an important role in substrate recognition and/or discrimination when Ago is interacting with a noncanonical siRNA-like substrate such as the DNA-substituted duplexes used in our study.

*Drosophila* RLC consisting of Dcr-2, Ago2 and R2D2 promotes siRNA loading (58). In the case of siRNA-based RNAi, guide strand selection appears carried out according to RNA duplex thermodynamics (52,56,57). Before loading onto Ago2, R2D2 binds to the more stable end, whereas Dcr-2 binds to the less stable end (50). Although Ago2/Dcr complexes associated with TRBP or PACT might be the primary complexes involved in RISC loading in humans (12), we are not yet sure about whether similar complexes play an essential role in strand selection in humans (59). Furthermore, at least for some siRNAs and miRNAs, Ago2 has been shown to be sufficient for loading and guide strand selection in the absence of Dcr (33–35). Note that the RNAi system depending on siRNA with a dsDNA seed duplex is required little, if any, in intracellular strand-selection mechanism described above, since the fate to become guide or passenger strands has been determined almost immediately after cell transfection (Figure 1B). Thus, our RNAi system due to DNA-modified siRNA might provide some important insight into Dcr-independent RNAi mechanism.

One of the most surprising findings in the present study may be that, although effective RNAi triggered by siRNA with a dsDNA seed duplex appeared to require TRBP activity similar, if not identical, to that due to nonmodified authentic siRNA, the former exhibited a much lower affinity to purified recombinant TRBP than the latter (see Figure 6C and Supplementary Figure S6C). The TRBP concentration giving 50% binding of siRNA with a dsDNA seed duplex was estimated at ∼50 μM (see Figure 6C and Supplementary Figure S6C), which roughly corresponds to one molecule in a sphere of 400 Å diameter, leading us to the notion that a functional intracellular TRBP should be associated with an unidentified RNA-binding RLC/RISC protein. One of the possible candidates may be Ago protein. However, as interaction between TRBP and Ago protein is conflicting, the other unidentified protein(s) ‘X’, which is a possible substitute for Dcr and stimulates the access of TRBP protein to siRNA as in the case of *Drosophila* R2D2/Dcr-2 complexes, might not be excluded [Figure 7B pre-binding step; (50)].

Human TRBP contains two dsRNA-binding domains, dsRBD1 and dsRBD2, both of which include α-β-β-β-α–folds and three amino acids presumably involved in dsRNA binding in any ∼16-nt region (60,61). Mutagenesis along with structural analysis indicated that each TRBP domain is capable of interacting with dsRNA via at least two sites in minor and major grooves, which are <11 nt apart from each other and close to the distance between the distal ends of siRNA subdomains A and C (see the upper margin in Figure 3). Furthermore, we showed both dsRBD1 and dsRBD2 to be necessary for effective TRBP binding to siRNA (51). In the dsRNA helix, a minor groove is located on the opposite-side surface of a major groove, and a major groove is located on the opposite-side surface of a minor groove. Thus, it may be suggested that two sets of major and minor grooves aligned with the non-seed-duplex region face-to-face so that two dsRBDs of TRBP can hold siRNA stably from both sides.

*Drosophila* R2D2 has been shown to be localized in the vicinity of the nucleotide at position 20 in the 3′ overhang of the guide strand. However, we are not yet sure about whether human TRBP is similarly capable of recognizing the 3′ overhang of the guide strand, although RNA in the 3′ terminal domain (subdomain T) of the guide strand may slightly increase TRBP binding (see Figure 6 and Supplementary Figure S6). The nucleotide duplex geometry may be important for TRBP binding to siRNA. TRBP can bind to A-form duplex but not to B-form duplex. So, it seems likely that the differences in affinity of TRBP for different types of DNA-substituted siRNAs may somewhat correlate with a gradual shift away from A-form duplex geometry toward B-form geometry according to the increase of DNA content.

Our data shown in Figure 2 indicate RNA-requirement–dependent subdomain structure in the non-seed-duplex region to be primarily due to that in the guide-strand sequence but not in the passenger-strand sequence in all cells examined, indicating differential involvement of siRNA guide and passenger strands in RNAi pathway. One might imagine that strand selection occurs at an early stage of RLC/RISC development so that the contribution of the passenger-strand to RNAi may be virtually completely excluded. However, this may not be the case. Except for the seed duplex region, nucleotides of the passenger strand as a duplex partner of the guide strand with a 2–3-bp DNA modification are all ribonucleotides and hence, all potential protein-binding sites on the passenger strand remain intact.

The passenger strand is eventually eliminated from the RISC (37) so that any information from the passenger strand should become dispensable from the time of unwinding of siRNA duplex or passenger elimination (mature RISC step in Figure 7B). Our data in Figure 5 indicated Ago strongly binds to the 3′ terminal subdomain T through the enhancer function due to the subdomain-C RNA sequence in the guide strand, thus indicating that guide and passenger strands have been correctly recognized at this stage of RLC/RISC development. We presume interactions between Ago-PAZ domain and siRNA subdomains (T+C) to occur at pre-RISC step in Figure 7B. Lane 11 in Figure 5 showed the presence of 5′ protruded RNA strand to prevent the PAZ domain of Ago protein from binding to the siRNA terminal subdomain and hence, PAZ of Ago may recognize the siRNA subdomain T, which is associated with 3′ RNA protrusion of the guide strand, as a binding site.

Knockdown of RLC/RISC-related gene activity (see Figure 4) clearly showed the involvement of both Ago2 and TRBP in RNAi under our experimental conditions. However, increment in relative IC_50_ due to knockdown of Ago2 was, in general, much higher than that due to knockdown of TRBP. Thus, in some cases, TRBP function may be replaced by other dsRNA binding protein such as PACT (see Figure 4C). However, PACT and TRBP do not always work in redundant manner; PACT in complex with Dcr is shown to inhibit the processing of pre-siRNA substrates when compared with Dcr and a Dcr-TRBP complex (37). Knockdown of TRBP may generate a state where Dcr preferentially binds to PACT, and inhibits the siRNA-promoting capability during initial substrate recognition, Dicer processing and possibly RISC loading. Alternatively, neither dsRNA binding protein nor presumed unidentified RLC/RISC protein, ‘X’, may be involved in our RNAi system. In this case, Ago2 is also a possible candidate of ‘X’, as *in vitro*, Ago2 is capable of binding to double-stranded siRNA, unwinding siRNA and incorporating the guide-strand to form an active mature RISC for silencing the intended target gene [see Figure 7B; (37)].

In conclusion, we used DNA replacement for the analysis of subdomain structures of siRNA not only in a previous study (55) but in the present study as well, and demonstrated that siRNA consists of a seed duplex and four non-seed-duplex subdomains, three of which are essential for Ago2 and TRBP binding.

## SUPPLEMENTARY DATA

Supplementary Data are available at NAR Online.

## FUNDING

This work was supported by the Ministry of Education, Culture, Sports, Science and Technology of Japan, Cell Innovation Program (MEXT) [21310123 and 21115004] and Core Research Project for Private University matching fund subsidy (to K.U.-T.) and a Research Fellowship for Young Scientists of the Japan Society for the Promotion of Science (to T.T.). Funding for open access charge: Research grant form Ministry of Education,Culture, Sports, Science and Technology of Japan [21115004 to K.U.-T.] and JSPS (to T.T.).

*Conflict of interest statement*. None declared.

## Supplementary Material

Supplementary Data
