# Supplementary material for: Interactions between the non-seed region of siRNA and RNA-binding RLC/RISC proteins, Ago and TRBP, in mammalian cells
Source: Nucleic Acids Res. 2014 Feb 20;42(8):5256–69. doi: 10.1093/nar/gku153 (PMC4005638; doi:10.1093/nar/gku153)
Supplement: Supplementary Data [file supp_42_8_5256__index.html]

Interactions between the non-seed region of siRNA and RNA-binding RLC/RISC proteins, Ago and TRBP, in mammalian cells — Interactions between the non-seed region of siRNA and RNA-binding RLC/RISC proteins, Ago and TRBP, in mammalian cells — Supplementary Data 

# Interactions between the non-seed region of siRNA and RNA-binding RLC/RISC proteins, Ago and TRBP, in mammalian cells

## Supplementary Data

files

**Files in this Data Supplement:**

- Supplementary Data - pdf file
