# Supplementary material for: Interactions between the non-seed region of siRNA and RNA-binding RLC/RISC proteins, Ago and TRBP, in mammalian cells
Source: Nucleic Acids Res. 2014 Feb 20;42(8):5256–69. doi: 10.1093/nar/gku153 (PMC4005638; doi:10.1093/nar/gku153)
Supplement: Supplementary Data [file supp_gku153_nar-00107-y-2014-File003.pdf]

Supplementary Figure S1.

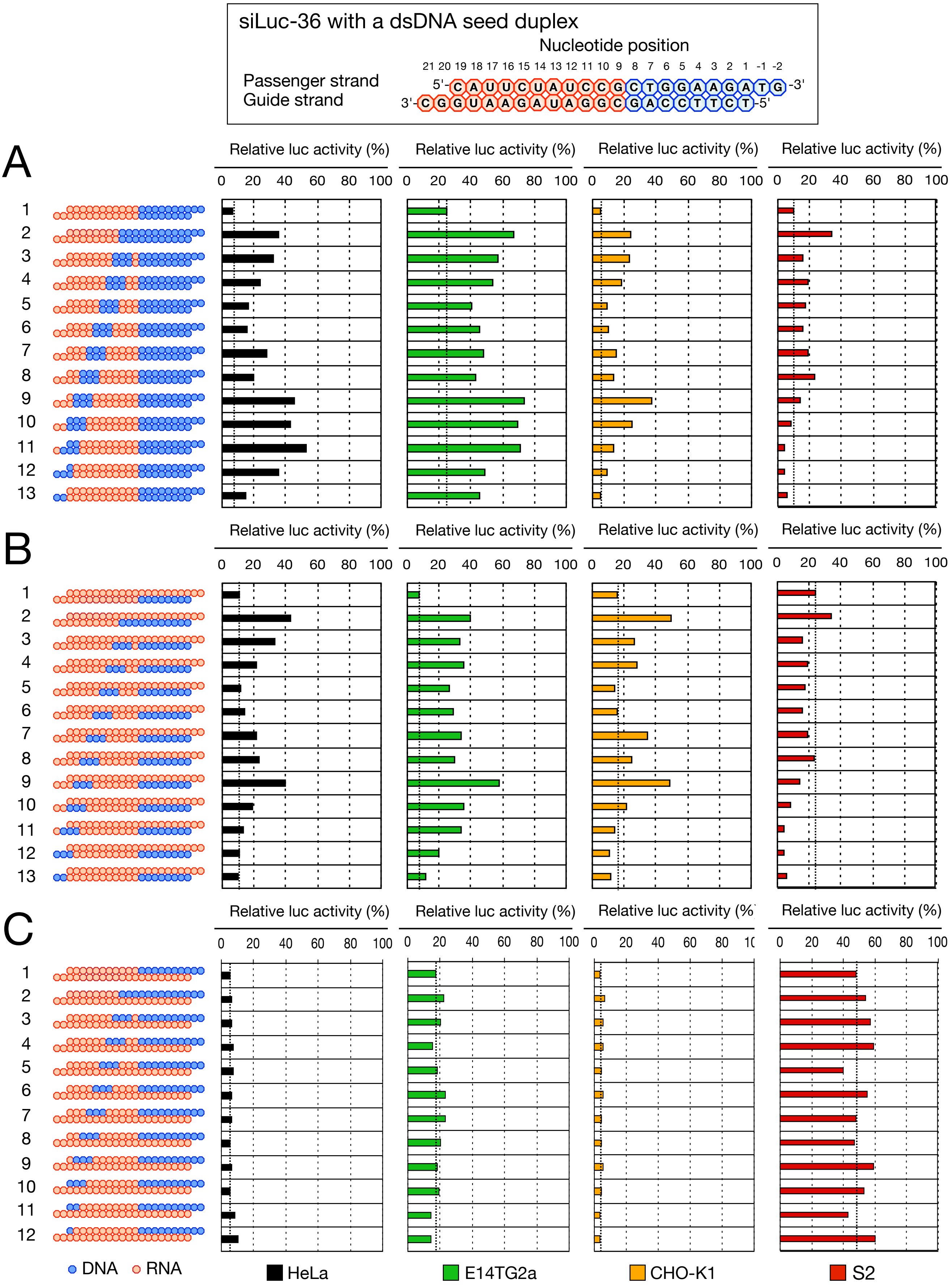

Supplementary Figure S1. Effects of 3bp-long DNA substitutions in the non-seed region of siRNA (siLuc-36) with a dsDNA seed duplex on RNAi activity as determined by *luc* reporter assays. Except for DNA/RNA difference, the nucleotide sequence of DNA-modified siRNAs used here were identical to that of siLuc-36. Red circle, ribonucleotide; Blue circle, deoxyribonucleotide. (**A**) Both guide and passenger strands were simultaneously replaced with DNA. (**B**) DNA replacement was carried out only in the guide strand. (**C**) DNA replacement was carried out only in the passenger strand. RNAi was assayed using human HeLa, mouse E14TG2a, Chinese hamster CHO-K1, and *Drosophila* S2 cells using a dual-luciferase reporter assay system with non-modified or DNA-substituted siRNAs at 50 nM. However, in S2 RNAi (B), the siRNA concentration used was 5nM. The dotted line in each graph indicates the level of RNAi due to the parental modified siRNA. Lane 1, siRNA with a dsDNA seed duplex. Lane 2-lane 13, siRNA with a dsDNA-modified seed duplex and two additionally DNA-substituted base pairs in the non-seed duplex region.

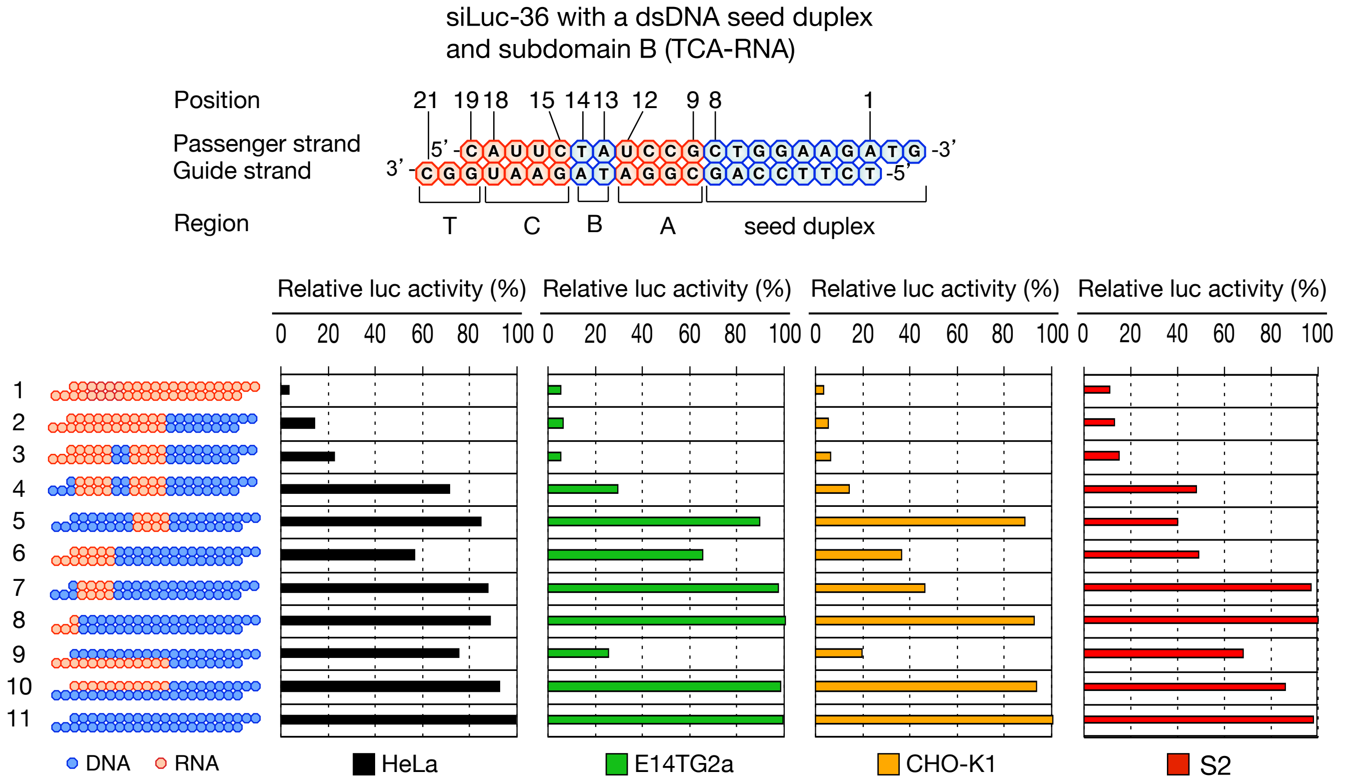

Supplementary Figure S2. Effects of DNA substitutions in the non-seed duplex subdomains A, B, C and T on RNAi activity. Except for DNA/RNA difference, the nucleotide sequences of DNA-modified siRNAs were identical to that of siLuc-36. Red circle, ribonucleotide; Blue circle, deoxyribonucleotide. Seed duplex, nucleotide position 2 to 8; subdomain A, position 9 to 12; subdomain B, position 13 to 14; subdomain C, position 15 to 18; subdomain T, position 19 to 21. RNAi activity was assayed using HeLa, E14TG2a, CHO-K1, and S2 cells, using a dual-luciferase reporter assay system. The concentration of siRNA was 50 nM. Lane 1, non-modified siRNA; lane 2, TCBA-RNA (the entire non-seed duplex is RNA); lane 3, TCA-RNA; lane 4, CA-RNA; lane 5, A-RNA; lane 6, TC-RNA; lane 7, C-RNA; lane 8, T-RNA; lane 9, RNA in the guide strand non-seed region; lane 10, RNA in the passenger non-seed region; lane 11, siDNA.

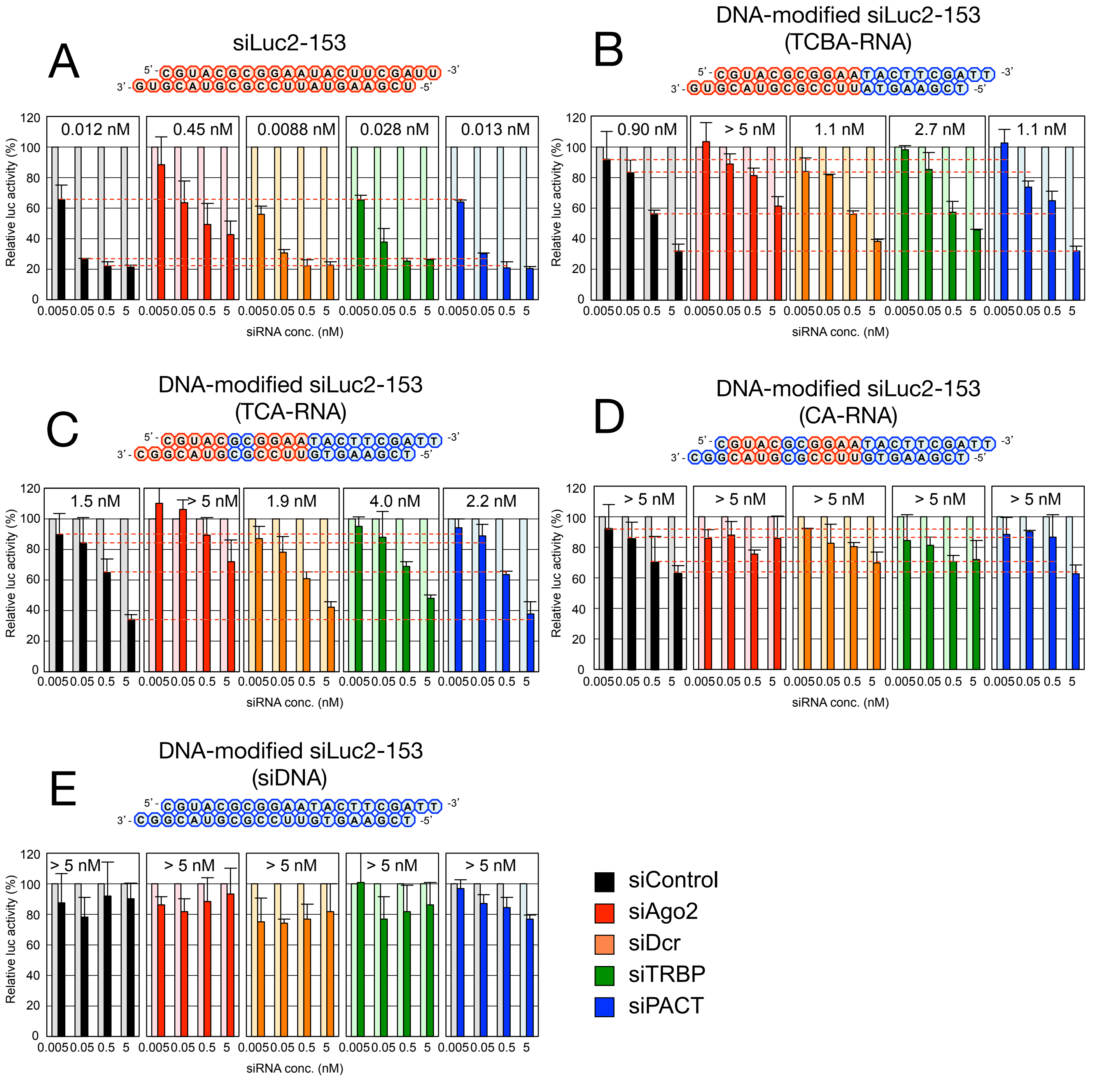

Supplementary Figure S3. Effects of Ago2, Dcr, TRBP, and PACT knockdown on RNAi activity of siLuc2-153 and its derivatives. RNAi activity was measured using a dual-luciferase reporter assay system using HeLa cells. siRNA structures are schematically shown in the upper margin. The concentration of anti-*luc* siRNA ranged from 5 pM to 5 nM, while those of siAgo2, siDcr, siTRBP, and siPACT were fixed at 25 nM. Calculated IC_{50} values were shown in the top of each panel. (A) Authentic or non-modified siRNA (siLuc2-153). (B) siLuc2-153 with a dsDNA seed duplex (TCBA-RNA). (C) siLuc2-153 with dsDNA-substituted seed duplex and subdomain B (TCA-RNA). (D) siLuc2-153 with dsDNA-substituted seed duplex and subdomains B and T (CA-RNA). (E) siDNA.

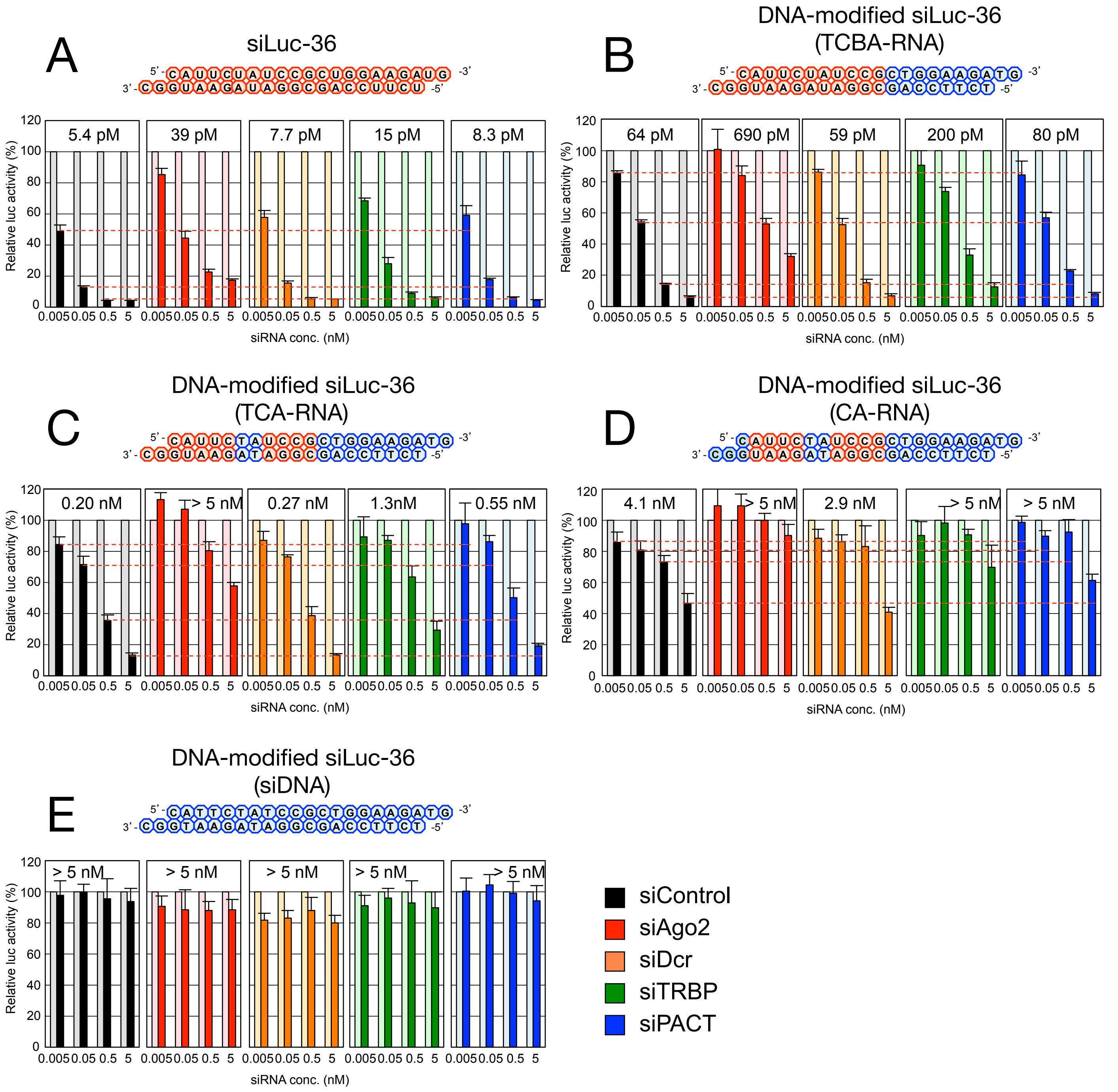

Supplementary Figure S4. Effects of Ago2, Dcr, TRBP, and PACT knockdown on RNAi activity of siLuc-36 and its derivatives. RNAi activity was measured using a dual-luciferase reporter assay system using HeLa cells. siRNA structures are shown in the upper margin of each panel. The concentration of anti-*luc* siRNA ranged from 5pM to 5 nM, while those of siAgo2, siDcr, siTRBP, and siPACT were fixed at 25 nM. Calculated IC_{50} values were shown in the top of each panel. (A) Authentic or non-modified siRNA (siLuc-36). (B) siLuc-36 with a dsDNA seed duplex (TCBA-RNA). (C) siLuc-36 with dsDNA-substituted seed duplex and subdomain B (TCA-RNA). (D) siLuc-36 with dsDNA-substituted seed duplex and subdomains B and T (CA-RNA). (E) siDNA.

PAZ domain

```
hAgo1   PVIEFMCEVLDIRNIDEQPKPLTDSQRVRFTKEIKGLKVEVTHCGQMKRKYRVCNVTRRPASHQTFPLQLESGQTVECTVAQYFKQKYNLQLKYPHLPCLQVGQEQKHTYLPLEVCNIV
hAgo2   PVIEFVCEVLDFKSIEEQQKPLTDSQRVKFTKEIKGLKVEITHCGQMKRKYRVCNVTRRPASHQTFPLQQESGQTVECTVAQYFKDRHKLVLRYPHLPCLQVGQEQKHTYLPLEVCNIV
hAgo3   PVIQFMCEVLDIHNIDEQPRPLTDSHRVKFTKEIKGLKVEVTHCGTMRRKYRVCNVTRRPASHQTFPLQLENGQTVERTVAQYFREKYTLQLKYPHLPCLQVGQEQKHTYLPLEVCNIV
hAgo4   PIIEFMCEVLDIQNINEQTKPLTDSQRVKFTKEIRGLKVEVTHCGQMKRKYRVCNVTRRPASHQTFPLQLENGQAMECTVAQYFKQKYSLQLKYPHLPCLQVGQEQKHTYLPLEVCNIV

mAgo1   PVIEFMCEVLDIRNIDEQPKPLTDSQRVRFTKEIKGLKVEVTHCGQMKRKYRVCNVTRRPASHQTFPLQLESGQTVECTVAQYFKQKYNLQLKYPHLPCLQVGQEQKHTYLPLEVCNIV
mAgo2   PVIEFVCEVLDFKSIEEQQKPLTDSQRVKFTKEIKGLKVEITHCGQMKRKYRVCNVTRRPASHQTFPLQQESGQTVECTVAQYFKDRHKLVLRYPHLPCLQVGQEQKHTYLPLEVCNIV
mAgo3   PVIQFMCEVLDIHNIDEQPRPLTDSHRVKFTKEIKGLKVEVTHCGTMRRKYRVCNVTRRPASHQTFPLQLENGQTVERTVAQYFREKYTLQLKYPHLPCLQVGQEQKHTYLPLEVCNIV
mAgo4   PIIEFMCEVLDIQNINEQTKPLTDSQRVKFTKEIRGLKVEVTHCGQMKRKYRVCNVTRRPASHQTFPLQLENGQAMECTVAQYFKQKYSLQLKHPHLPCLQVGQEQKHTYLPLEVCNIV

chAgo1  PVIEFMCEVLDIRNIDEQPKPLTDSQRVRFTKEIKGLKVEVTHCGQMKRKYRVCNVTRRPASHQTFPLQLESGQTVECTVAQYFKQKYNLQLKYPHLPCLQVGQEQKHTYLPLEVCNIV
chAgo2  PVIEFVCEVLDFKSIEEQQKPLTDSQRVKFTKEIKGLKVEITHCGQMKRKYRVCNVTRRPASHQTFPLQQESGQTVECTVAQYFKDRHKLVLRYPHLPCLQVGQEQKHTYLPLEVCNIV
chAgo3  PVIQFMCEVLDIHNIDEQPRPLTDSHRVKFTKEIKGLKVEVTHCGTMRRKYRVCNVTRRPASHQTFPLQLENGQTVERTVAQYFREKYALQLKYPHLPCLQVGQEQKHTYLPLEVCNIV
chAgo4  PIIEFMCEVLDIQNINEQTKPLTDSQRVKFTKEIRGLKVEVTHCGQMKRKYRVCNVTRRPASHQTFPLQLENGQAMECTVAQYFKQKYSLQLKYPHLPCLQVGQEQKHTYLPLEVCNIV
```

Supplementary Figure S5. Amino acid sequence alignments of the PAZ domains of Ago1~4 from human, mouse and Chinese hamster. Blue, amino acid residues conserved in all 12 Agos; Green, amino acid residues conserved in three of four Agos in each animal.

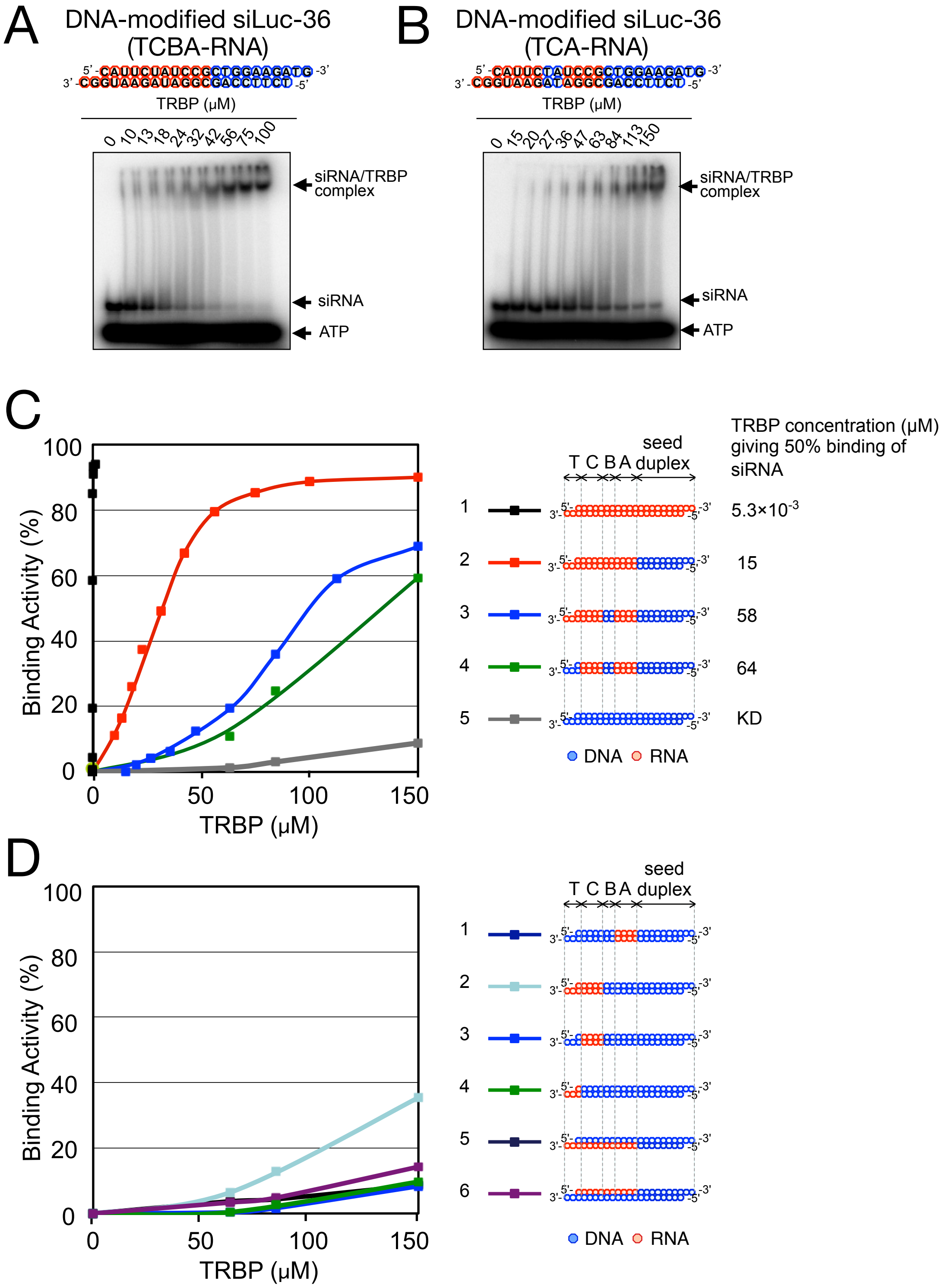

Supplementary Figure S6. EMSA of DNA-substituted siRNAs associated with recombinant human TRBP protein. Except for DNA/RNA difference, the nucleotide sequence of DNA-modified siRNA was identical to that of siLuc-36. Red circle, ribonucleotide; Blue circle, deoxyribonucleotide. (A,B) EMSA experiments. Purified recombinant TRBP and ^{32}P-labeled siRNA with DNA substitutions (0.5 nM) were incubated with increasing amounts of wild-type TRBP protein, as indicated. In both (A) and (B), seed duplex region was replaced with dsDNA. In (B), dsDNA substitution was also carried out in subdomain B. (C, D) A bound fraction (%) of siRNA was plotted against the input concentration of TRBP. Note that the binding activities of DNA-modified siRNAs were significantly lower than that of the non-modified siRNA. Structures of DNA modifications are shown in the right margin (C, D). In (C), observed TRBP concentrations giving 50% binding of non-modified siRNA and siRNA with a dsDNA seed duplex are also shown in the right margin.

Supplementarty Figure S7.

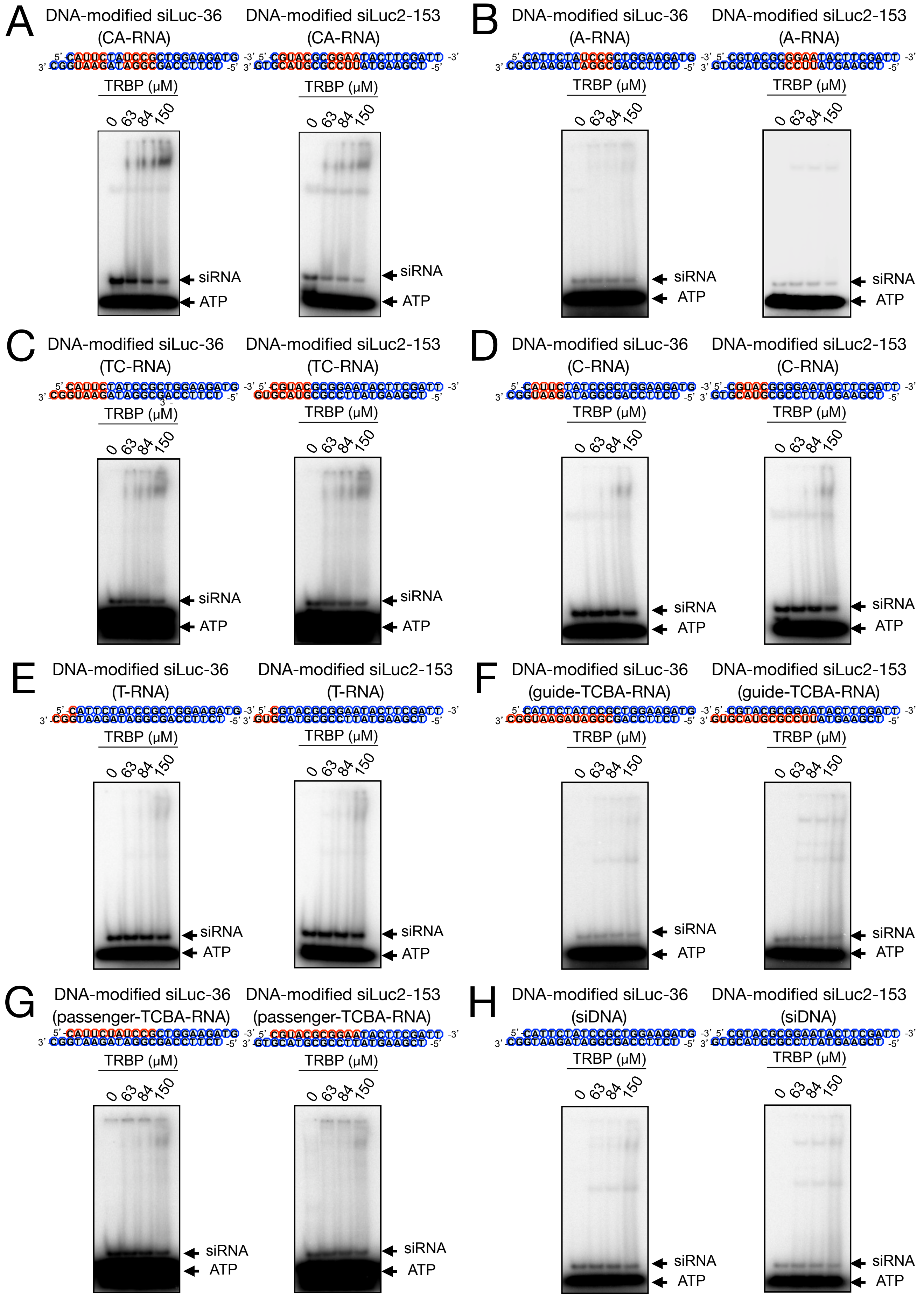

Supplementary Figure S7. EMSA profiles of DNA-substituted siRNAs with recombinant human TRBP protein. Results from EMSA experiments using recombinant human TRBP with ^{32}P-labeled DNA-modified siLuc-36(CA-RNA) and siLuc2-153(CA-RNA) (A), DNA-modified siLuc-36 (A-RNA) and siLuc2-153 (A-RNA) (B), DNA-modified siLuc-36 (TC-RNA) and siLuc2-153 (TC-RNA) (C), DNA-modified siLuc-36 (C-RNA) and DNA-modified siLuc2-153 (C-RNA) (D), DNA-modified siLuc-36 (T-RNA) and DNA-modified siLuc2-153 (T-RNA) (E), DNA-modified siLuc-36 (RNA only in the passenger-strand non-seed region) and DNA-modified siLuc2-153 (RNA only in the passenger-strand non-seed region) (F),DNA-modified siLuc-36 (RNA only in the guide-strand non-seed region) and DNA-modified siLuc2-153 (RNA only in the guide-strand non-seed region) (G), and DNA-modified siLuc-36 (siDNA) and DNA-modified siLuc2-153 (siDNA) (H).
